# Reduced Expression of GPX3 in Breast Cancer Patients in Correlation with Clinical Significance

**DOI:** 10.1055/s-0040-1722170

**Published:** 2020-12-26

**Authors:** Pensri Saelee, Tanett Pongtheerat, Thanet Sophonnithiprasert

**Affiliations:** 1 Division of Research, National Cancer Institute, Bangkok, Thailand; 2 Unit of Biochemistry, Department of Medical Sciences, Faculty of Science, Rangsit University, Patumthani, Thailand

**Keywords:** breast cancer, GPX3, antioxidant enzyme, gene expression, real-time reverse transcription-PCR

## Abstract

Glutathione peroxidase 3 (GPX3) is the main antioxidant enzyme in plasma. Its biological roles are to protect cells from oxidative stress-induced damage. Several studies have been reported the association between GPX3 expression and its correlation with cancer carcinogenesis including breast cancer. The aim of this research was to investigate the GPX3 messenger ribonucleic acid (mRNA) expression in 82 breast tumors and paired normal breast tissues by SYBR green quantitative real-time reverse transcription-polymerase chain reaction and the association with clinicopathological data. Our results show that GPX3 reduced expression was found significantly associated with number of metastatic lymph nodes (odds ratio [OR] = 3.41, 95% confidence interval [CI] = 1.35–8.64, *p* = 0.01), no distant metastasis (OR = 5.52, 95% CI = 3.74–11.89, *p* = 0.04), and nonhormone usage breast cancer patients (OR = 0.19, 95% CI = 0.04–0.93, *p* = 0.04). This finding suggested that GPX3 plays a role in breast carcinogenesis, and might serve as a prognostic biomarker in breast cancer patients.

## Introduction

Glutathione peroxidase 3 (GPX3), a tumor suppressor gene that is located on chromosome 5q23, is the major antioxidant enzyme in plasma and plays an important role in detoxifying hydrogen peroxide and other oxygen-free radicals, protecting cell from oxidative stress-induced damage. 1 2 3

Inactivation of GPX3 results in the accumulation of an elevated amount of hydrogen peroxide and other reactive oxygen species (ROS) that may involve breast carcinogenesis via induction of oxidative deoxyribonucleic acid (DNA) damage, genetic alterations, and neoplastic transformation. 4 5 Several studies have reported the association between GPX3 expression and its correlation with cancer carcinogenesis such as gastric cancer, 3 6 cervical cancer, 7 thyroid cancer, 8 nonsmall cell lung cancer, 2 prostate cancer, 9 hepatocellular carcinoma (HCC), 10 including breast cancer 11 12 however, the mechanisms in breast tumorigenesis remain unclear.

Previously, our data from microarray analysis (data not shown) was identified for GPX3 expression, which is one of the consistently downregulated genes in breast tumor. In the current study, we studied the role of GPX3 in breast carcinogenesis by determined GPX3 mRNA expression in 82 breast tumors and paired normal breast tissues by SYBR green quantitative real-time reverse transcription-polymerase chain reaction (RT-PCR) and correlation with clinicopathological characteristics including overall survival.

## Materials and Methods

### Tumor Specimens

Eighty-two breast tumors and corresponding normal breast tissues were collected from the National Cancer Institute, Bangkok, Thailand, during the period 2007 to 2011. This study was approved by the Institutional Review Board of the National Cancer Institute, Bangkok, Thailand. Invasive ductal breast carcinoma patients who had not undergone chemotherapy or radiotherapy were recruited into this study. Tissue samples were preserved in RNA later reagent, (Ambion; Carlsbad, California, United States) and kept at –80°C until used. Patients' clinicopathological data such as age at diagnosis, tumor size, histological grade, axillary lymph-node status, number of lymph nodes, staging, triple-negative tumor (estrogen receptor [ER], progesterone receptor [PR], and human epidermal growth factor receptor 2 [HER2]), immunohistochemistry staining of ER, PR, and HER2, treatment (anthracycline and anthracycline + taxane), metastasis (lung, bone, and liver), hormone usage, cancer family history, alcohol consumption, and smoking status were collected from patient files.

### RNA Preparation and cDNA Synthesis

Total RNA was extracted from 82 breast tumors and their corresponding normal breast tissues using Trizol reagent, according to the instruction manual (Invitrogen; Carlsbad, California, United States). Oligotex mRNA purification kit (Qiagen; Hilden, Germany) was used for mRNA purification. The iScriptTM Select cDNA Synthesis Kit (Bio-Rad Laboratories, Inc.; Hercules, California, United States) was used to transcribe mRNA to cDNA synthesis using for RT-PCR (Invitrogen; Carlsbad, California, United States).

### GPX3 mRNA Expression Analysis by SYBR Quantitative Real-Time Reverse Transcription-PCR

Alterations in GPX3 mRNA expression levels were analyzed by LightCycler Instrument (Roche Diagnostics GmbH, Mannheim, Germany). The reaction components were 20 ng of template cDNA, 1x LightCycler FastStart DNA Master SYBR Green I (Roche Diagnostics GmbH, Mannheim, Germany), 4 mM MgCl _2_ and 0.5 μM forward and reverse primers in 10 μL of a total volume. The primer sequences were designed by Primer-BLAST, forward F-GPX3 (5′-AGTGCTGGACAGTGACAACC-3′) and reverse R-GPX3 (5′- GGCCCCAAGGTTGAGGTATC-3′. β-globin housekeeping gene was used as an endogenous reference to obtain relative expression values. PCR was started at 95°C for 5 minutes (to activate the FastStart Taq), followed by 40-cycle amplification (95°C for 10 seconds, 62°C for 30 seconds, and 72°C for 30 seconds. After the PCR, each amplification reaction was checked using a dissociation curve. PCR product purity was checked by 1.5% agarose gel electrophoresis, stained with ethidium bromide, and photographed under UV light. Relative gene expression level was determined as previously described by Livak and Schmittgen. 13 Median expression levels were used for the cutoff values for gene expression that were adopted from median expression levels. Gene expression <0.3 was assigned as reduced expression.

### Statistical Analysis

The association between GPX3 mRNA reduced expression level and clinicopathological characteristics–age at diagnosis; tumor size; histological grade; axillary lymph-node status; number of lymph nodes; staging; triple-negative breast tumor; immunohistochemistry staining of ER, PR, and HER2; treatment; metastasis; hormone usage; cancer family history; alcohol consumption; and smoking status–was examined statistically by chi-squared test. Overall survival was analyzed by Kaplan–Meir method and *p* -value < 0.05 was considered a significant correlation.

## Results

### Reduced Expression of GPX3 mRNA in Breast Cancer Patients

Previously, our data from microarray analysis was identified for GPX3 expression, which is one of the consistently downregulated genes. In this research, we verified GPX3 mRNA expression level in 82 breast tumors and corresponding normal breast tissues by SYBR quantitative real-time RT-PCR. The results show that GPX3 reduced expression was detected in 41.50% (34/82) that is consistent with microarray data that shown downregulated gene. In addition, we also found that GPX3 reduced expression was significantly associated with number of metastatic lymph nodes (more than 2 lymph nodes), (OR = 3.41, 95% CI = 1.35–8.64, *p* = 0.01) and no distant metastasis (OR = 5.52, 95% CI = 3.74–11.89, *p* = 0.04, Table 1 ). Moreover, GPX3 reduced expression and patients' clinical data of hormonal usage, cancer family history, alcohol consumption, and smoking status were analyzed. It was found that GPX3 reduced expression was associated with nonhormone usage breast cancer patients (OR = 0.19, 95% CI = 0.04–0.93, *p* = 0.04) as shown in Table 2 .

**Table 1:** Association between GPX3 reduced expression and clinicopathological data of 82 breast cancer patients by SYBR quantitative real-time reverse transcription-polymerase chain reaction

		GPX3 reduced expression			
Clinical data	*n*	GPX3–	GPX3+	Odds ratio (95%CI)	*p* -Value
		*n* (%)	*n* (%)		
Age				1.45 (0.59–3.49)	0.50
≤ 50	43	27 (56.3)	16 (47.0)		
> 50	39	21 (43.7)	18 (53.0)		
Tumor size(cm)				2.07 (0.51–8.44)	0.35
≤ 2	11	8 (16.7)	3 (8.8)		
> 2	71	40 (83.3)	31 (91.2)		
Histologic grade				2.22 (0.77–6.44)	0.82
I + II	45	27 (56.3)	18 (52.9)		
III	37	21 (43.7)	16 (47.1)		
Tumor stage				1.52 (0.63–3.70)	0.37
I, IIA, IIB	46	29 (60.4)	17 (50.0)		
IIIA, IIIB	36	19 (39.6)	17 (50.0)		
Lymph-node status				1.20 (0.48–2.99)	0.82
Negative	31	19 (39.6)	12 (35.3)		
Positive	51	29 (60.4)	22 (64.7)		
Number of lymph nodes				3.41 (1.35–8.64)	0.01
0–2 positive	50	35 (72.9)	15 (44.1)		
> 2 positive	32	13 (27.1)	19 (55.9)		
Immunohistochemical					
ER status				1.53 (0.58–4.07)	0.47
Negative	25	16 (35.6)	9 (26.5)		
Positive (1 + ,2 + ,3 + )	54	29 (64.4)	25 (73.5)		
PgR status				1.69 (0.68–4.18)	0.36
Negative	36	23 (51.1)	13 (38.2)		
Positive (1 + ,2 + ,3 + )	43	22 (48.9)	21 (61.8)		
HER2 status				1.03 (0.38–2.73)	1.00
Negative	56	32 (71.1)	24 (70.6)		
Positive (1 + ,2 + ,3 + )	23	13 (28.9)	10 (29.4)		
Triple negative tumor				0.55 (0.17–1.78)	0.40
ER, PR, HER2 positive	62	34 (75.6)	28 (84.8)		
ER, PR, HER2 negative	16	11 (24.4)	5 (15.2)		
Treatment				3.00 (0.99–9.01)	0.06
Anthracycline	39	27 (75.0)	12 (50.0)		
Anthracycline + taxane	21	9 (25.0)	12 (50.0)		
Distant metastasis				5.52 (3.74–11.89)	0.04
No	47	34 (82.9)	13 (56.5)		
Yes	17	7 (17.1)	10 (43.5)		

Abbreviations: –, no reduced expression; +, reduced expression; CI, confidence interval; GPX3, glutathione peroxidase 3; HER2, human epidermal growth factor receptor 2; ER, estrogen receptor; PR, progesterone receptor.

**Table 2:** Association between GPX3 reduced expression and clinical data of hormone usage, cancer family history, alcohol consumption, and smoking status of 81 breast cancer patients by SYBR quantitative real-time reverse transcription-polymerase chain reaction

		GPX3 reduced expression			
Clinical data	*n*	GPX3–	GPX3+	Odds ratio (95%CI)	*p* -Value
		*n* (%)	*n* (%)		
Hormone usage				0.19 (0.04–0.93)	0.04
Never	67	36 (75.0)	31 (93.9)		
Ever	14	12 (25.0)	2 (6.1)		
Cancer family history				0.20 (0.02–1.75)	0.15
No	47	29 (78.4)	18 (94.7)		
Yes	9	8 (21.6)	1 (5.3)		
Alcohol consumption				0.39 (0.13–1.22)	0.12
Never	61	33 (68.7)	28 (84.8)		
Ever	20	15 (31.3)	5 (14.7)		
Smoking status					
Never	78	46 (95.8)	32 (97.0)	0.72 (0.06–8.27)	1.00
Ever	3	2 (4.2)	1 (3.0)		

Abbreviations: –, no reduced expression; +, reduced expression; CI, confidence interval; GPX3, glutathione peroxidase 3.

### Survival Analysis

**Fig. 1:**
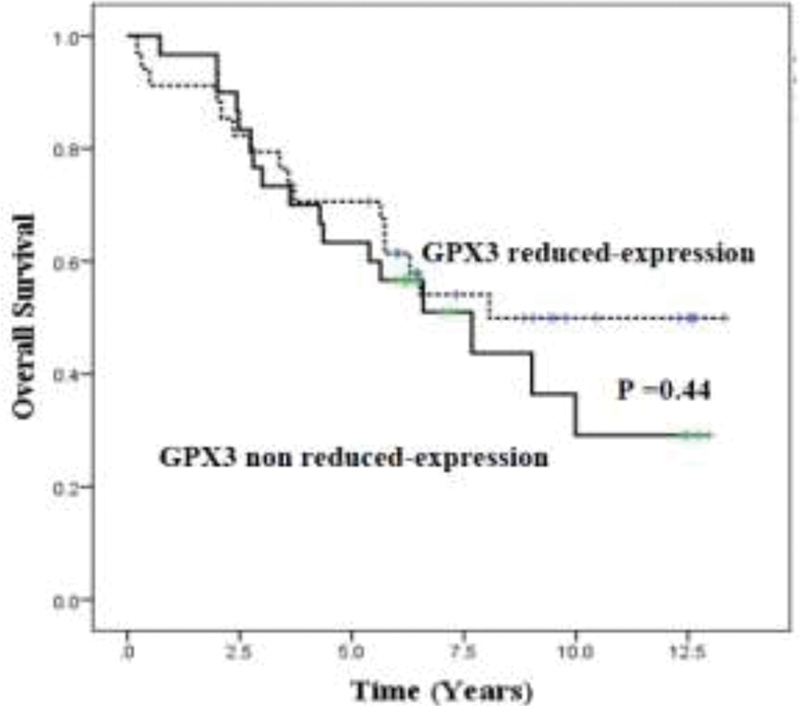
Survival was analyzed by Kaplan–Meir method and log rank test was used to compare between glutathione peroxidase 3 reduced expression and nonreduced expression, *p* = 0.44.

The association between GPX3 reduced expression and survival was analyzed by Kaplan–Meir method. The results show that there was no correlation between GPX3 reduced expression and overall survival ( *p* = 0.44) as shown in Fig. 1 .

## Discussion

Glutathione peroxidase (GPx) is a major antioxidative damage enzyme family, which comprises eight submembers (GPx 1–8). GPX3 is the only extracellular enzyme in the GPx family that removing ROS products during cellular metabolism or oxidative damage. 1 14

GPX3 has been reported to be downregulated in several types of cancers such as GPX3 downregulation that can promote the proliferation, motility, and invasion of melanoma cells in vitro. 15 Qi et al found that GPX3 low expression was significantly associated with advanced tumor stage, venous infiltration, and poor overall survival in HCC patients 10 as well as in gallbladder cancer 16 that has been shown poor prognosis.

Furthermore, Mohamed et al 11 demonstrated that downregulation of GPx3 levels was found in aggressive inflammatory breast cancer carcinoma tissues when comparing to noninflammatory breast cancer tissues as well as Lou et al 12 reported that GPX3 mRNA and protein expression level in breast cancer tissues was expressed less than corresponding normal tissues, suggesting the involvement of GPX3 in breast pathogenesis.

In the present study, we found that GPX3 reduced expression correlated with number of metastatic lymph nodes (more than 2 lymph nodes) ( *p* = 0.01), as in accordance with previous research, the downregulation of GPX3 expression was significantly associated with lymph node metastasis in gastric cancer and cervical cancer 3 6 7 as well as reduced GPX3 protein levels were correlated with tumor size and lymph node metastasis in thyroid cancer. 8 In addition, several studies has been reported GPX3 downregulation was promoted tumor invasion, motility. 10 15 However, our findings were found that reduced expression of GPX3 was significantly relevant to no distant metastasis in breast cancer patients ( *p* = 0.04); this demonstrated the uncomplete inactivation of GPX3 expression was found in this study. Furthermore, we analyzed the association between GPX3 reduced expression and clinical data of hormone usage, cancer family history, alcohol consumption, and smoking status; we also found on the first time that GPX3 reduced expression was significantly correlated with nonhormone usage in breast cancer patients ( *p* = 0.04).

In conclusion, our finding showed that GPX3 reduced expression was significantly correlated with number of metastatic lymph nodes, no distant metastasis, and nonhormone usage of breast cancer patients; this finding suggested that GPX3 plays an important role in breast carcinogenesis, and might serve as a prognostic biomarker in breast cancer patients.

## References

[1] Multidisciplinary Research Group on Diabetes of the Instituto Mexicano del Seguro Social Baez-Duarte B G Mendoza-Carrera F García-Zapién A Glutathione peroxidase 3 serum levels and GPX3 gene polymorphisms in subjects with metabolic syndrome Arch Med Res 2014 45 05 375 382 24819036 10.1016/j.arcmed.2014.05.001

[2] Liu K Jin M Xiao L Liu H Wei S Distinct prognostic values of mRNA expression of glutathione peroxidases in non-small cell lung cancer Cancer Manag Res 2018 10 10 2997 3005 30214294 10.2147/CMAR.S163432 PMC6118261

[3] Min S Y Kim H S Jung E J Jung E J Jee C D Kim W H Prognostic significance of glutathione peroxidase 1 (GPX1) down-regulation and correlation with aberrant promoter methylation in human gastric cancer Anticancer Res 2012 32 08 3169 3175 22843889

[4] Ambrosone C B Oxidants and antioxidants in breast cancer Antioxid Redox Signal 2000 2 04 903 917 11213491 10.1089/ars.2000.2.4-903

[5] Cebrian A Pharoah P D Ahmed S Tagging single-nucleotide polymorphisms in antioxidant defense enzymes and susceptibility to breast cancer Cancer Res 2006 66 02 1225 1233 16424062 10.1158/0008-5472.CAN-05-1857

[6] Peng D F Hu T L Schneider B G Chen Z Xu Z K El-Rifai W Silencing of glutathione peroxidase 3 through DNA hypermethylation is associated with lymph node metastasis in gastric carcinomas PLoS One 2012 7 10 e46214 23071548 10.1371/journal.pone.0046214 PMC3468580

[7] Zhang X Zheng Z Yingji S Downregulation of glutathione peroxidase 3 is associated with lymph node metastasis and prognosis in cervical cancer Oncol Rep 2014 31 06 2587 2592 24788695 10.3892/or.2014.3152

[8] Zhao H Li J Li X Silencing GPX3 expression promotes tumor metastasis in human thyroid cancer Curr Protein Pept Sci 2015 16 04 316 321 25929866 10.2174/138920371604150429154840

[9] Yu Y P Yu G Tseng G Glutathione peroxidase 3, deleted or methylated in prostate cancer, suppresses prostate cancer growth and metastasis Cancer Res 2007 67 17 8043 8050 17804715 10.1158/0008-5472.CAN-07-0648

[10] Qi X Ng K T Lian Q Z Clinical significance and therapeutic value of glutathione peroxidase 3 (GPx3) in hepatocellular carcinoma Oncotarget 2014 5 22 11103 11120 25333265 10.18632/oncotarget.2549 PMC4294380

[11] Mohamed M M Sabet S Peng D F Nouh M A El-Shinawi M El-Rifai W Promoter hypermethylation and suppression of glutathione peroxidase 3 are associated with inflammatory breast carcinogenesis Oxid Med Cell Longev 2014 2014 787195 24790704 10.1155/2014/787195 PMC3980917

[12] Lou W Ding B Wang S Fu P Overexpression of GPX3, a potential biomarker for diagnosis and prognosis of breast cancer, inhibits progression of breast cancer cells in vitro Cancer Cell Int 2020 20 378 32782436 10.1186/s12935-020-01466-7 PMC7412804

[13] Livak K J Schmittgen T D Analysis of relative gene expression data using real-time quantitative PCR and the 2(-Delta Delta C(T)) Method Methods 2001 25 04 402 408 11846609 10.1006/meth.2001.1262

[14] Jiao Y Wang Y Guo S Glutathione peroxidases as oncotargets Oncotarget 2017 8 45 80093 80102 29108391 10.18632/oncotarget.20278 PMC5668124

[15] Chen H Zheng Z Kim K Y Jin X Roh M R Jin Z Hypermethylation and downregulation of glutathione peroxidase 3 are related to pathogenesis of melanoma Oncol Rep 2016 36 05 2737 2744 27600457 10.3892/or.2016.5071

[16] Yang Z L Yang L Zou Q Positive ALDH1A3 and negative GPX3 expressions are biomarkers for poor prognosis of gallbladder cancer Dis Markers 2013 35 03 163 172 24167362 10.1155/2013/187043 PMC3774968

